# Decoupling the Dual Impact of NISQ Noise on Quantum Adversarial Robustness

**DOI:** 10.3390/e28070719

**Published:** 2026-06-24

**Authors:** Haoran Wang, Shaoliang Ye, Shaowei Wang, Hanyi Wang, Zhenbo Shi, Wei Yang

**Affiliations:** 1 School of Computer Science and Technology, University of Science and Technology of China, Hefei 230026, China; 2 Suzhou Institute for Advanced Research, University of Science and Technology of China, Suzhou 215123, China; 3 Hefei National Laboratory, University of Science and Technology of China, Hefei 230088, China; 4 School of Artificial Intelligence, Guangzhou University, Guangzhou 510006, China; 5 China Mobile (Suzhou) Software Technology Co., Ltd., Suzhou 215000, China

**Keywords:** quantum machine learning, quantum adversarial attacks, variational quantum classifier, attack transferability

## Abstract

As quantum machine learning modules become increasingly integrated into NISQ-era infrastructures, it remains unclear whether intrinsic device noise can be regarded as a passive defense against adversarial examples, or whether it in fact introduces a new attack surface. To answer this question, we propose a noise-aware four-path evaluation protocol that decouples the noise assumed at attack generation from the noise present at inference, and we systematically test it on a 4-qubit variational quantum classifier over four datasets with depolarizing probabilities in the range p∈[0,0.3], using both standard gradient attacks and expectation over transformation (EOT)-based attacks. The results show that for some datasets, higher noise does suppress attacks, whereas for others attacks remain effective even at p=0.3, and in several cases a moderate noise level even maximizes the attack success rate. Moreover, we find that adversarial examples generated under moderate noise often attack the clean model more successfully than those generated in an ideal setting, demonstrating that noise can be actively exploited by an adversary to discover more transferable adversarial directions. Therefore, ambient noise should not be treated as a built-in security guarantee, and future quantum machine learning (QML) robustness evaluations must explicitly model such noise-aware threats.

## 1. Introduction

Secure quantum communication and networked quantum infrastructures are moving from laboratory prototypes toward practical systems for key establishment, authenticated control, and distributed sensing [1,2,3]. At the same time, core quantum-cryptographic primitives, especially quantum key distribution, are now mature enough that their performance and security must be evaluated in realistic and heterogeneous environments [4,5,6]. Future infrastructures may incorporate quantum or quantum-assisted learning modules for network monitoring, state classification, and device-anomaly detection, raising the question of how such modules behave under adversarial conditions.

The hardware on which many of these modules are expected to run is still in the noisy intermediate-scale quantum (NISQ) regime, where gate imperfections and readout errors cannot be ignored [7]. At the same time, quantum machine learning (QML) has shown that parameterized quantum circuits and variational models can act as compact learners for small and medium data regimes [8,9,10]. For security-supporting tasks that must run on limited quantum resources, this makes such models particularly attractive.

Typical constructions, such as variational quantum classifiers (VQCs) with data re-uploading or feature-space encodings, provide expressive decision boundaries while keeping circuit depth low [11,12]. This combination of NISQ hardware and variational models is a plausible candidate for near-term quantum-network and security-oriented applications.

From the classical side, however, it is well established that learned models can be driven to misclassify by adversarial examples, i.e., inputs that are imperceptibly but carefully perturbed [13,14,15]. Adaptive strategies such as expectation over transformation (EOT) were designed precisely for randomized or noisy inference: by averaging the loss over multiple stochastic forward passes, an attacker can still recover a usable gradient through a non-deterministic model [16]. This is particularly relevant for quantum models, where measurement outcomes and device noise are inherently stochastic. This motivates the question of whether quantum classifiers embedded in quantum-cryptographic or quantum-network stacks inherit similar fragility when the queried model is noisy, making EOT-style attacks particularly relevant.

Recent research has given an initial answer. Lu, Duan, and Deng [17] demonstrated that quantum classifiers are vulnerable to adversarial perturbations in much the same way as classical neural networks, and that this holds both for classical data fed to quantum circuits and for genuinely quantum data. Liu and Wittek [18] analyzed the geometry of quantum classification and showed that small perturbations in the encoded state can already trigger label changes, especially in higher-dimensional Hilbert spaces. Building on these foundations, Gong and Deng [19] studied universal adversarial perturbations for quantum classifiers and showed that a single data-agnostic perturbation can fool a wide range of quantum models, confirming that the phenomenon is not related to a specific circuit instance [20]. Most of these studies, however, generated and evaluated attacks on idealized simulators or under very simple noise assumptions, so we still lack a controlled understanding of how strength-varying simulated noise can suppress adversarial effectiveness or reshape it. On the defense side, Huang et al. [21] showed that explicitly inserting controllable noise layers into quantum neural networks can improve their adversarial robustness via a min–max optimization, highlighting that noise can also be engineered as part of the defense. Later benchmarking work on robust QML started to systematize attack–defense evaluations, but it still concentrated on clean or only lightly perturbed scenarios, leaving out realistic hardware-level and strength-varying noise conditions [22,23].

What is largely missing from these studies is a careful treatment of how explicitly modeled noise changes adversarial evaluation protocols. Most quantum adversarial works generate attacks on ideal simulators and then evaluate on the same, effectively assuming a clean quantum channel. In practice, however, depolarizing and readout errors are ubiquitous, and there is now a mature line of work on error mitigation and certification whose main message is that such noise cannot be dismissed [24,25,26]. This leads to a security-relevant and nontrivial question: Does device noise automatically weaken adversarial examples, so that noise can be regarded as a free defense, or does this effect only appear in a limited range of noise strengths and placements? For future quantum infrastructures that may combine cryptographic protocols with QML-based monitoring or classification, answering this question is a necessary preliminary step before any claim can be made about relying on ambient noise as a security mechanism.

In this paper, we focus on one controlled noise configuration: independent single-qubit depolarizing channels inserted immediately before measurement in a variational quantum classifier running on a noisy simulator. We focus on two concrete questions. First, when adversarial examples are generated on a clean model, how does their attack success rate evolve as the depolarizing probability increases, and how does this change depend on whether the classifier is evaluated with or without noise-matched retraining? Second, when adversarial examples are generated on the corresponding noisy model using an EOT-style strategy, do they become more effective on the same noisy model and more transferable back to the clean model?

The contributions of this paper are threefold. First, we propose a noise-aware four-path evaluation protocol for quantum adversarial attacks. The protocol separates clean-generated/clean-inference, clean-generated/noisy-inference, noisy-generated/noisy-inference, and noisy-generated/clean-inference paths, allowing inference-side noise and generation-side noise awareness to be analyzed separately. Second, we compare standard gradient attacks with EOT-based noise-aware attacks on simulated noisy variational quantum classifiers, in order to examine whether apparent robustness under noisy inference persists against adaptive attackers. Third, we extend the study across several classical benchmarks reduced to four-qubit inputs and construct a cross-noise transferability matrix, revealing that adversarial examples generated at intermediate noise levels can transfer better across heterogeneous noisy models than attacks generated on a perfectly clean simulator.

The remainder of this paper is organized as follows. Section 2 reviews the NISQ noise models and the fundamental concepts of variational quantum classifiers. Section 3 formulates the security-oriented adversarial robustness problem under noisy inference scenarios. Section 4 details the evaluation procedure and the noise-aware attack methods. Section 5 describes the experimental setup. Section 6 reports and analyzes the experimental results. Section 7 concludes the paper and discusses future work.

## 2. Preliminaries

In this section, we set notation and recall the quantum machine learning ingredients used in the rest of the paper. We briefly review parameterized quantum circuits and their use in VQCs, the data-encoding scheme, the NISQ-oriented noise models, and the threat model for quantum adversarial examples.

### 2.1. Parameterized Quantum Circuits and VQCs

Let $H={\left(C^{2}\right)}^{⊗n}$ be the Hilbert space of *n* qubits. A parametrized quantum circuit (PQC) is a unitary of the form

(1) $$U\left(\theta \right)=U_{L}\left(\theta_{L}\right)\cdots U_{2}\left(\theta_{2}\right)U_{1}\left(\theta_{1}\right)$$

where each $U_{𝓁}\left(\theta_{𝓁}\right)$ is obtained from a fixed gate (or block of gates) and a real parameter $\theta_{𝓁}\in R$. Given an input state $\rho_{\mathrm{in}}$ and a PQC U(θ), a variational model produces an output state

(2) $$\rho_{\theta }=U\left(\theta \right)\;\rho_{\mathrm{in}}\;U^{†}\left(\theta \right)$$

which is then measured. For binary classification, a common choice is to measure one qubit in the computational basis and interpret the outcome as the class label, or to measure an observable *M* and apply a classical decision rule sign(〈M〉).

A variational quantum classifier is obtained by optimizing the circuit parameters θ on a labeled training set. Concretely, for training data $\left\{\left(x_{i},y_{i}\right)\right\}$, with $y_{i}\in \left\{0,1\right\}$, we define a model prediction

(3) $$f_{\theta }\left(x_{i}\right)=\mathrm{Tr}\left(M\;U\left(\theta \right)\;\rho \left(x_{i}\right)\;U^{†}\left(\theta \right)\right)$$

and a loss $L\left(\theta \right)=\frac{1}{N}\sum_{i}𝓁\left(f_{\theta }\left(x_{i}\right),y_{i}\right)$, where *ℓ* is, for example, the squared loss or a margin-based loss. Optimization is carried out on a classical computer using gradient-based or gradient-free routines.

### 2.2. Quantum Data Encoding

In order to process classical data with a PQC, one needs to map a classical feature vector $x\in R^{d}$ to a quantum state ρ(x). Several encoding strategies are standard:

- Angle encoding: each feature $x_{j}$ is used as a rotation angle of a single-qubit gate, e.g.,
  (4) $$\rho \left(x\right)=\overset{m}{\underset{j=1}{⨂}}\left(R_{Y}\left(x_{j}\right)|0\rangle \langle 0|R_{Y}^{†}\left(x_{j}\right)\right)$$
  possibly preceded or followed by entangling layers.
- Amplitude encoding: *x* is normalized to unit ${𝓁}_{2}$ norm and loaded directly into the amplitudes of an *n*-qubit state,
  (5) $$\rho \left(x\right)=|x\rangle \langle x|,\;|x\rangle =\sum_{k}x_{k}|k\rangle$$
  This achieves compact representations at the cost of more involved state preparation.

In the remainder of this work, we assume an angle-encoding scheme, which is commonly supported by NISQ-oriented frameworks followed by a trainable PQC. The exact choice of encoding does not affect the definitions below, as long as the map x↦ρ(x) is fixed.

### 2.3. Noise Models for NISQ Devices

Current quantum processors are unavoidably noisy. A convenient way to describe this is through completely positive and trace-preserving (CPTP) maps acting on single or two qubits. In line with widely used simulators such as PennyLane, we consider:

- Depolarizing noise. A single-qubit depolarizing channel with strength p∈[0,1] acts as
  (6) $$D_{p}\left(\rho \right)=\left(1-p\right)\rho +\frac{p}{3}\left(X\rho X+Y\rho Y+Z\rho Z\right)$$
  where X,Y,Z are the Pauli operators. This models isotropic gate errors.
- Amplitude damping. This channel describes energy relaxation with probability γ:
  (7) $$A_{\gamma }\left(\rho \right)=E_{0}\rho E_{0}^{†}+E_{1}\rho E_{1}^{†}$$
  where $E_{0}=|0\rangle \langle 0|+\sqrt{1-\gamma }|1\rangle \langle 1|$ and $E_{1}=\sqrt{\gamma }|0\rangle \langle 1|$.
- Measurement/readout error. A classical bit-flip channel acting on the measurement outcome, typically parameterized by $p_{0\to 1}$ and $p_{1\to 0}$, to account for misread results.

In a gate-level simulation, such channels can be inserted after each one- or two-qubit gate, or immediately before the measurement. For a circuit U(θ) executed under a noise configuration N, we denote by

(8) $$E_{\theta }^{N}\left(\rho \right)=N\circ U_{\theta }\left(\rho \right)$$

the overall noisy evolution, where $U_{\theta }\left(\rho \right)=U\left(\theta \right)\rho U^{†}\left(\theta \right)$ and N is the composition of the relevant CPTP maps. Observables are then evaluated on $E_{\theta }^{N}\left(\rho \left(x\right)\right)$.

### 2.4. Parameter-Shift Gradient

Because gates in VQCs are typically of the form $e^{-i\frac{\theta }{2}P}$, with *P* a Pauli operator or tensor product of Pauli operators, expectation values are differentiable with respect to circuit parameters and can be computed using the parameter-shift rule [10]:

(9) $$\frac{𝜕}{𝜕\theta }\langle M\rangle =\frac{1}{2}({\langle M\rangle }_{\theta +\frac{\pi }{2}}-{\langle M\rangle }_{\theta -\frac{\pi }{2}})$$

where ${\langle M\rangle }_{\theta \pm \frac{\pi }{2}}$ denotes the expectation of *M* when the circuit parameter is shifted by ±π/2. This rule allows one to estimate gradients using only circuit evaluations, and it remains applicable in the presence of Markovian noise when the same shift is applied to the noisy circuit.

### 2.5. Threat Model and Quantum Adversarial Examples

We consider a white-box setting in which the attacker knows the circuit structure and the trained circuit parameters. In the noise-aware attacks, the attacker is also assumed to know the noise family and the exact depolarizing probability *p* used during attack generation. The attack uses the same noise distribution and finite-shot sampling model as the corresponding inference setting, but not the same individual random draws. Let *x* be a clean classical input, and let *y* be its true label. The VQC with parameters θ and noise configuration N induces a prediction

(10) $$\hat{y}=g\left(E_{\theta }^{N}\left(\rho \left(x\right)\right)\right)$$

where g(·) denotes the classical post-processing of measurement results such as thresholding a single expectation value. A quantum adversarial example for *x* is another input $\tilde{x}$, constrained to be close to *x* under a chosen metric in the classical feature space, such that

(11) $$g\left(E_{\theta }^{N}\left(\rho \left(\tilde{x}\right)\right)\right)\neq y$$

The attacker’s objective is thus to solve

(12) $$\max_{\tilde{x}\in B\left(x\right)}\;J\left(E_{\theta }^{N}\left(\rho \left(\tilde{x}\right)\right),y\right)$$

where B(x) is a small neighborhood of *x*, for example an ${𝓁}_{p}$ ball in the encoded classical space, and J is a loss function that increases when the model misclassifies the input. When N=0, which corresponds to the noiseless case, (12) reduces to the optimization problem considered in earlier work on quantum adversarial classification [17,18]. When N≠0, the same form allows one either to evaluate how noise alone affects adversarial examples, or to optimize through the noise by estimating gradients with the parameter-shift rule.

The definitions above do not presuppose any particular dataset, circuit size, or noise strength, and they will be used in the sequel to define several evaluation paths, namely clean to clean, clean to noisy, noisy to noisy and noisy to clean.

## 3. Problem Formulation

This section formalizes the setting for studying how quantum noise affects adversarial examples on variational quantum classifiers. We first specify the model and noise assumptions, then state two research questions, and finally introduce the four-path evaluation protocol that mirrors our experiments.

### 3.1. Model and Noise Setting

Let $H={\left(C^{2}\right)}^{⊗n}$ be the Hilbert space of *n* qubits. A VQC is specified by a parameterized circuit U(θ) and a fixed measurement observable *M*, for example the Pauli-*Z* operator on the first qubit. For an input $x\in R^{d}$, a classical-to-quantum encoding map x↦ρ(x) prepares the input state, and the classifier outputs

(13) $$f_{\theta }\left(x\right)=\mathrm{Tr}\;\left(M\;U\left(\theta \right)\rho \left(x\right)U^{†}\left(\theta \right)\right)$$

with a subsequent classical decision rule $g(f_{\theta }\left(x\right))\in \left\{0,1\right\}$. In NISQ conditions, the circuit does not act as a perfect unitary. Instead, execution is followed by a CPTP map $N_{p}$ that models depolarizing, amplitude-damping, or readout errors with strength parameter p≥0. We denote the noisy model by

(14) $$f_{\theta }^{N_{p}}\left(x\right)=\mathrm{Tr}\;\left(M\;N_{p}\left(U\left(\theta \right)\rho \left(x\right)U^{†}\left(\theta \right)\right)\right)$$

In experiments, we sweep *p* over p∈{0,0.02,0.05,0.10,0.20,0.30} to emulate different NISQ noise levels. For p=0, we recover the ideal simulator. We consider two training modes:

- Clean-trained model: θ is obtained by minimizing a supervised loss on $f_{\theta }$ in the noiseless setting (13).
- Noise-trained model: For a given p>0, $\theta_{p}$ is obtained by minimizing the same loss but with $f_{\theta }^{N_{p}}$ in (14). This matches the common practice of retraining under the target noise model to compensate for accuracy loss at higher *p*.

In the main experiments, the clean-trained model is used for p=0, while the noise-trained model $\theta_{p}$ is used for each nonzero *p*. Clean-weight reuse is treated only as an auxiliary diagnostic mode.

### 3.2. Adversarial Objective

Given a correctly classified input (x,y), y∈{0,1}, an adversarial example is another input $\tilde{x}$ in a prescribed perturbation set B(x) such that the classifier mislabels it. For the noiseless model, the attacker solves

(15) $$\max_{\tilde{x}\in B\left(x\right)}\;L\left(f_{\theta }\left(\tilde{x}\right),y\right)$$

where L is a monotone loss. When the attacker is aware that inference will run under $N_{p}$, a natural objective is the following noise-aware expectation variant:

(16) $$\max_{\tilde{x}\in B\left(x\right)}\;E_{\omega ∼N_{p}}\left[L\left(f_{\theta }^{N_{p},\omega }\left(\tilde{x}\right),y\right)\right]$$

where $f_{\theta }^{N_{p},\omega }$ denotes one stochastic execution of the noisy circuit. During adversarial example generation, the trained circuit parameters θ are kept fixed, and only the input $\tilde{x}$ is updated. The expectation is estimated by averaging over independently sampled noisy executions with the same depolarizing probability *p* and the same shot setting as inference. Gradients are therefore averaged over repeated executions of the same parameterized circuit, rather than over different model parameters [27].

### 3.3. Research Questions

The above setting gives rise to two concrete questions that we aim to answer experimentally:

#### 3.3.1. RQ1: Noise as Conditional Defense

When adversarial examples are generated on a noiseless model using (15), how does their attack success rate (ASR) change as a function of (i) the noise strength *p*, (ii) whether the model was trained cleanly or under noise. In particular, we ask whether there exists a range of *p* in which hardware-like noise degrades adversarial effectiveness more than it degrades clean accuracy, as suggested by prior observations on noise-induced robustness [28,29].

#### 3.3.2. RQ2: Noise-Aware Attack

When adversarial examples are generated using the noise-aware objective (16) under the same noise model used at inference, does this restore or even improve the ASR on noisy models, and does such an attack remain effective on the corresponding clean model? This question captures whether an adaptive adversary can take back the apparent robustness arising from noise.

### 3.4. Four-Path Evaluation Protocol

To answer RQ1 and RQ2 in a single experimental sweep over *p*, we evaluate each attack under the following four paths:

1. Path A (clean-gen → clean): Adversarial examples are generated on the clean model $f_{\theta }$ and evaluated on the same clean model. Path A serves as the clean-inference reference for the same model instance.
2. Path B (clean-gen → noisy): The same adversarial examples from Path A are evaluated on the noise-matched model $f_{\theta_{p}}^{N_{p}}$. The ASR drop from A to B measures the passive defensive effect of noise at level *p*.
3. Path C (noisy-gen → noisy): Adversarial examples are generated on the noisy model using (16), that is, the attacker optimizes through the noise, and then evaluated on the same noisy model. The ASR gain from B to C shows how much of the passive defense disappears once the attacker is noise-aware.
4. Path D (noisy-gen → clean): Adversarial examples from Path C are fed back to the clean model $f_{\theta }$. This distinguishes noise-aware attacks that genuinely improve perturbation quality from those that merely overfit a specific noisy configuration.

Formally, let $A_{\mathrm{clean}}$ denote an attack algorithm that solves (15) approximately on $f_{\theta }$, and let $A_{\mathrm{noisy},p}$ denote an attack that solves (16) on $f_{\theta_{p}}^{N_{p}}$. For a test set ${\left\{\left(x_{i},y_{i}\right)\right\}}_{i=1}^{N}$, we define the per-path ASR as

(17) $$\begin{array}{cc} {\mathrm{ASR}}_{A}\left(p\right)\; & =\frac{1}{N_{c}}\sum_{i|g\left(f_{\theta }\left(x_{i}\right)\right)=y_{i}}1\left[g\left(f_{\theta }\left(A_{\mathrm{clean}}\left(x_{i}\right)\right)\right)\neq y_{i}\right] \end{array}$$

(18) $$\begin{array}{cc} {\mathrm{ASR}}_{B}\left(p\right)\; & =\frac{1}{N_{c}}\sum_{i|g\left(f_{\theta }\left(x_{i}\right)\right)=y_{i}}1\left[g\left(f_{\theta_{p}}^{N_{p}}\left(A_{\mathrm{clean}}\left(x_{i}\right)\right)\right)\neq y_{i}\right] \end{array}$$

(19) $$\begin{array}{cc} {\mathrm{ASR}}_{C}\left(p\right)\; & =\frac{1}{N_{c,p}}\sum_{i|g\left(f_{\theta_{p}}^{N_{p}}\left(x_{i}\right)\right)=y_{i}}1\left[g\left(f_{\theta_{p}}^{N_{p}}\left(A_{\mathrm{noisy},p}\left(x_{i}\right)\right)\right)\neq y_{i}\right] \end{array}$$

(20) $$\begin{array}{cc} {\mathrm{ASR}}_{D}\left(p\right)\; & =\frac{1}{N_{c,p}}\sum_{i|g\left(f_{\theta_{p}}^{N_{p}}\left(x_{i}\right)\right)=y_{i}}1\left[g\left(f_{\theta }\left(A_{\mathrm{noisy},p}\left(x_{i}\right)\right)\right)\neq y_{i}\right] \end{array}$$

Here $N_{c}$ denotes the samples correctly classified by the clean model, and $N_{c,p}$ those correctly classified by the noisy model at level *p*. In the actual experiments, the above procedure is repeated over a range of noise strengths *p*. In the main-text experiments, for every nonzero *p*, the model is retrained under the matched noise level before executing the four paths. This lets us identify a weak-noise region where ${\mathrm{ASR}}_{B}\left(p\right)<{\mathrm{ASR}}_{A}\left(p\right)$, meaning that noise appears to act as a defense, and a region where ${\mathrm{ASR}}_{C}\left(p\right)$ rises back toward ${\mathrm{ASR}}_{A}\left(p\right)$, meaning that noise can be exploited.

## 4. Methodology

Building on Section 3, this section describes the experimental protocol used in the study. We specify the variational circuit, the angle-encoding scheme, the depolarizing-noise layer, the clean and noise-aware attacks, and the four path-wise evaluations over different noise strengths. The protocol is designed to be reproducible in current NISQ simulation frameworks and follows the structure of the released implementation.

### 4.1. Circuit Architecture and Data Encoding

**Figure 1:**
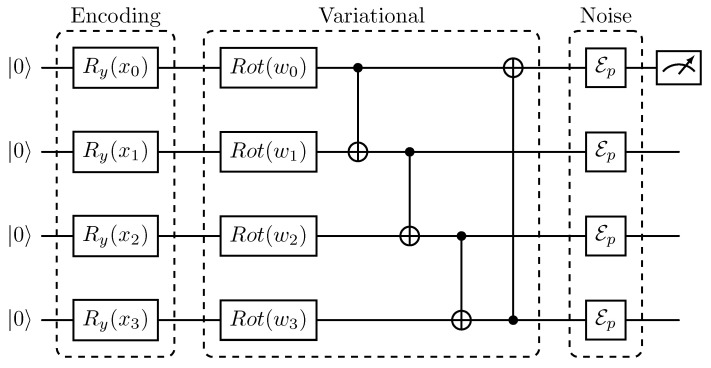
Quantum neural network architecture with an explicit noise layer.

We focus on compact variational quantum classifiers with n=4 qubits as a controlled proof-of-concept setting. Although this scale is smaller than many target NISQ applications, it is sufficient for the four-dimensional benchmark representations used here and makes exhaustive evaluation over noise levels, attack paths, and random seeds computationally feasible. The experimental circuit diagram is shown in Figure 1.

Each circuit has the following structure:

1. Angle encoding: A real-valued feature vector $x\in R^{4}$ is mapped to a quantum state by applying single-qubit rotations $R_{Y}\left(x_{j}\right)$ on qubits j=0,…,3. This choice is compatible with many classical datasets after standardization and rescaling to [−π,π].
2. Trainable entangling layers: We stack one or more layers of parameterized two-qubit entangling blocks. In our implementation we use the StronglyEntanglingLayers pattern, which alternates single-qubit rotations and controlled entangling gates over all wires. The trainable parameters of these layers form the vector θ.
3. Measurement: The expectation value of *Z* on the first qubit is measured and mapped to a class label by thresholding at 0.5 after affine rescaling.

This template is deliberately simple: it enables gradient-based attacks, supports a fixed depolarizing layer inserted before measurement, and keeps the circuit depth within the regime where mixed-state simulation remains tractable.

### 4.2. Noise Injection and Training Modes

The main experiments use a single controlled noise model. Specifically, we insert independent single-qubit depolarizing channels of strength *p* immediately before measurement. Concretely, for each qubit *w* we apply

(21) $$D_{p}\left(\rho \right)=\left(1-p\right)\rho +\frac{p}{3}\left(X\rho X+Y\rho Y+Z\rho Z\right),$$

so that the output state of the trainable circuit passes through $D_{p}^{⊗n}$ prior to measurement. This placement provides a tunable late-stage noise abstraction that is close to the measurement interface, preserves a clear separation between attack-generation noise and inference noise, and keeps the four-path evaluation computationally tractable. In Section 6 we vary *p* over

p∈{0.0,0.02,0.05,0.1,0.2,0.3},

which interpolates from the ideal simulator to a clearly noise-dominated regime.

For the main results, we use noise-matched retraining. That is, for each nonzero *p*, we train a separate VQC with the same architecture and with the corresponding depolarizing channel active during training, obtaining parameters $\theta_{p}$. This reflects a calibrated setting in which the model parameters are adapted to the expected noise level before adversarial evaluation.

### 4.3. Adversarial Attacks

We instantiate the clean attack $A_{\mathrm{clean}}$ and the noise-aware attack $A_{\mathrm{noisy},p}$ with standard gradient-based methods adapted to quantum circuits [30].

Given a correctly classified input (x,y), we compute the gradient of the loss with respect to the encoded input angles. Since each classical feature is implemented as an $R_{Y}\left(x_{i}\right)$ rotation, this gradient is the derivative with respect to the corresponding encoding gate parameter. In implementation, the trained model parameters are fixed and PennyLane’s differentiable QNode interface is used to obtain $\nabla_{x}L$. We then update *x* inside an ${𝓁}_{\infty }$ ball of radius ε:

(22) $$x^{\left(t+1\right)}=\Pi_{x,\varepsilon }\left(x^{\left(t\right)}+\alpha \;\mathrm{sign}\left(\nabla_{x}L\left(f_{\theta }\left(x^{\left(t\right)}\right),y\right)\right)\right)$$

where $\Pi_{x,\varepsilon }$ denotes projection back to the admissible perturbation set and α is the step size. For t=0 we obtain a fast gradient sign method (FGSM); for t>1 we obtain projected gradient descent (PGD). These are the attacks used along Paths A and B.

In our implementation, depolarizing noise is represented as a CPTP map in a mixed-state simulator. Thus, the noisy state evolution itself is deterministic at the density-matrix level. Stochasticity enters through finite-shot measurement: with 1024 shots, each circuit execution returns a sampled estimate of the noisy expectation value. We therefore use EOT to average over finite-shot noisy executions of the same circuit. For each attack step, the trained circuit parameters and the noise probability *p* are kept fixed, and we compute K=16 input-gradient estimates from independent finite-shot executions before applying the PGD update. Formally,

(23) $$\nabla_{x}\tilde{L}=\frac{1}{K}\sum_{k=1}^{K}\nabla_{x}L\left(f_{\theta }^{N_{p},\omega_{k}}\left(x\right),y\right)$$

where $\omega_{k}$ denotes the *k*-th independent finite-shot execution of the same noisy circuit, not a different set of model parameters. For each $\omega_{k}$, the input gradient is computed using the same differentiable QNode and parameter-shift-compatible differentiation with respect to the encoded input angles. The *K* gradients are averaged first, and the averaged gradient is then used in the FGSM, PGD, or momentum update. This attack is used in Paths C and D. If analytic deterministic density-matrix outputs are used instead of finite shots, the CPTP map does not by itself generate independent stochastic realizations; in that case EOT reduces to repeated evaluations of the same noisy expectation.

### 4.4. Path-Wise Evaluation over Noise Levels

For each attack type and each noise level *p*, we execute the four evaluation paths defined in Section 3:

1. generate adversarial examples on the clean model and evaluate them on the clean model (Path A);
2. reuse these examples on the noisy model at level *p* (Path B);
3. generate adversarial examples directly on the noisy model at level *p* using the EOT variant (Path C);
4. test these noisy-generated adversarial examples back on the clean model (Path D).

At every step we record the ASR defined there, along with the average ${𝓁}_{2}$ norm of the input gradients. Repeating this procedure for all *p* yields four ASR curves

$${\mathrm{ASR}}_{A}\left(p\right),\;{\mathrm{ASR}}_{B}\left(p\right),\;{\mathrm{ASR}}_{C}\left(p\right),\;{\mathrm{ASR}}_{D}\left(p\right),$$

which can be plotted on the same axes. The gap ${\mathrm{ASR}}_{A}\left(p\right)-{\mathrm{ASR}}_{B}\left(p\right)$ identifies the range where noise acts as a passive defense; the gap ${\mathrm{ASR}}_{C}\left(p\right)-{\mathrm{ASR}}_{B}\left(p\right)$ quantifies how much of that passive defense can be neutralized by an adaptive adversary; and the behavior of ${\mathrm{ASR}}_{D}\left(p\right)$ indicates whether the noise-aware perturbations genuinely improve attack strength or merely overfit a particular noisy configuration.

In the sequel we specify the datasets, hyperparameters and simulators used to instantiate this procedure.

## 5. Experiments

**Figure 2:**
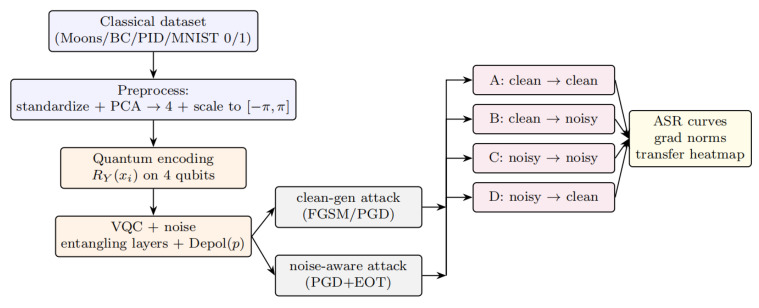
Overall pipeline of the proposed NISQ-oriented adversarial evaluation.

This section describes the experimental setup used to assess the effect of a controlled measurement-before-readout depolarizing noise model on quantum adversarial attacks. All experiments were carried out with the PennyLane-based implementation described in the accompanying code, which supports multiple classical datasets, a fixed 4-qubit variational classifier, several gradient-based attacks, and noise scanning over a depolarizing channel. The experimental pipeline is shown in Figure 2.

### 5.1. Datasets

To ensure that the observations are not tied to a single data distribution, we consider four binary classification problems, all represented by four real-valued features so that they can be encoded on four qubits. For datasets with more than four original features, we reduce the inputs to four principal components before angular encoding. This choice follows from the 4-qubit angle-encoding design, but it also changes the geometry of the original feature space. Therefore, the adversarial robustness results should be interpreted in the reduced encoded space:

- Synthetic: The two-moons dataset (make_moons) with 400 samples, noise level 0.15, standardized and zero-padded to 4 dimensions, then linearly mapped to [−π,π] for quantum encoding. This dataset creates a nonlinearly separable boundary and is useful to test whether adversarial examples remain effective when the circuit must learn such a boundary.
- Breast cancer (BC): The scikit-learn breast cancer dataset, originally derived from the UCI Machine Learning Repository, contains 569 samples and 30 features. It was standardized, reduced by principal component analysis (PCA) to 4 principal components, and then mapped to [−π,π]. Labels are benign and malignant. This dataset represents a low-noise, real biomedical classification task.
- Pima Indians Diabetes (PID): The OpenML diabetes task (768 samples, 8 features) was standardized, reduced to 4 dimensions (PCA) and mapped to [−π,π], with tested_positive as label 1. This dataset has moderate class imbalance and is useful to see how noise interacts with a less separable task.
- MNIST (0/1): MNIST was restricted to digits 0 and 1, standardized and reduced to 4 principal components, then mapped to [−π,π]. This provides a higher-dimensional source distribution collapsed to a 4D quantum input.

Table 1 summarizes the datasets used in the experiments.

For all datasets, a stratified split was used with 70% for training and 30% for testing. To reduce sensitivity to a particular initialization or data split, each experiment was repeated over five independent random seeds. Unless otherwise stated, we report the mean ASR and one standard deviation across seeds.

### 5.2. Quantum Model and Devices

All experiments used a 4-qubit variational quantum classifier. Each input vector $x\in R^{4}$ is encoded by applying $R_{Y}\left(x_{i}\right)$ on qubit *i*, followed by a single instance of StronglyEntanglingLayers acting on all four wires, which provides trainable one-qubit rotations and entangling gates in a hardware-efficient pattern. The model outputs the expectation value of *Z* on the first qubit. This circuit structure follows common VQC practice for NISQ-compatible classification and is identical across clean and noisy runs. Clean circuits are evaluated with a state-vector simulator, while noisy circuits are evaluated with a mixed-state simulator that supports CPTP noise channels. All reported experiments use 1024 shots. The depolarizing channel is applied deterministically at the density-matrix level, and stochasticity enters through finite-shot measurement sampling during training, inference, and attack generation. The detailed configuration is shown in Table 2.

### 5.3. Noise Configuration

As a controlled abstraction of late-stage hardware imperfections, we insert an independent single-qubit depolarizing channel with probability *p* on every wire immediately before measurement. We scan six noise levels,

p∈{0.0,0.02,0.05,0.10,0.20,0.30},

covering weak, moderate, and strong depolarizing regimes within the simulator. This range allows us to test whether increasing channel noise monotonically suppresses adversarial effectiveness, or whether intermediate noise levels can reshape the decision boundary in a way that remains exploitable by adaptive attacks [31].

For p=0, we train a clean model. For each nonzero *p*, our main experiments use noise-matched retraining: the VQC is trained with the same depolarizing channel active and then evaluated under that target noise level. This setting reflects a calibrated deployment in which the model parameters are adapted to the expected hardware noise. The clean-weight reuse mode is kept as an auxiliary option for isolating pure inference-time noise.

### 5.4. Attack Configurations

We consider four gradient-based adversarial attacks, implemented directly on the quantum model, as shown in Table 3:

- FGSM with perturbation budget ε=0.3 on the encoded features, after which the perturbed input is projected back to [−π,π] component-wise. This projection is necessary because each feature is ultimately realized as a rotation angle in $R_{Y}\left(\cdot \right)$, and keeping it within a single 2π-period avoids degeneracies due to the periodicity of the gate. The rest follows the classical prescription in [13].
- PGD with step size α=0.02, 10 iterations and the same budget ε=0.3, projected at every step to the same ${𝓁}_{\infty }$ box and to [−π,π] to remain in the valid encoding range; this is the basic iterative attack used in classical robustness studies.
- PGD-EOT, where each input gradient is estimated by averaging over K=16 independent noisy evaluations of the loss gradient at the current adversarial iterate, using the same trained circuit parameters and the same generation-side depolarizing probability *p*. This variant is the main tool to test whether a noise-aware attacker can recover attack success on noisy quantum channels.
- Momentum PGD-EOT, which adds a momentum term to stabilize noisy gradients, again with K=16 EOT samples drawn from the same noise distribution. This variant represents a stronger adaptive attack that tests whether momentum-stabilized gradients can further improve attack success under noisy evaluation.

All attacks are performed in the encoded angular feature space after preprocessing. We use a fixed perturbation budget ε=0.3 for all datasets so that the four evaluation paths are compared under the same attack constraint. Since the original data distributions and preprocessing transformations differ across datasets, this budget is not intended to represent the same semantic perturbation magnitude in the original feature space. Accordingly, the reported ASR values are used mainly for path-wise comparisons within each dataset, while cross-dataset differences are interpreted as task-dependent behavior under a common encoded-space attack budget.

All attacks are run on the test split only. Clean inputs are first evaluated to determine the subset of correctly classified examples; ASR is then computed only on this subset, i.e.,

(24) $$\mathrm{ASR}=\frac{\left|\{\;x|f\left(x\right)=y,\;f\left(x_{\mathrm{adv}}\right)\neq y\;\}\right|}{\left|\{\;x|f(x)=y\;\}\right|}$$

where f(·) denotes the classifier evaluated under the specified inference noise level.

### 5.5. Four-Path and Cross-Noise Experiments

To separate the effect of where the attack is generated from where it is evaluated, we run the following four canonical paths:

1. clean-generated → clean-inference (baseline vulnerability);
2. clean-generated → noisy-inference (apparent noise-induced defense);
3. noisy-generated → noisy-inference (noise-aware attacker);
4. noisy-generated → clean-inference (overfitting to a specific noise level).

This is implemented in the code by training models at each *p*, generating adversarial examples on the chosen source noise, and then re-evaluating them on the target noise.

In addition, we construct a cross-noise transferability matrix: for every pair $\left(p_{\mathrm{gen}},p_{\mathrm{infer}}\right)$ in the above noise set, we generate attacks at $p_{\mathrm{gen}}$ using the noise-aware PGD-EOT variant and measure ASR at $p_{\mathrm{infer}}$. The result is an N×N matrix of ASR values, saved as a heatmap, which directly answers whether there exists an intermediate attack noise level that transfers best across heterogeneous noisy models.

### 5.6. Training and Implementation Details

Unless otherwise stated, all models are trained for 120 optimization steps with mini-batches of size 16 and learning rate 0.08 using the PennyLane gradient-descent optimizer. This configuration is found to be empirically sufficient for convergence on all four datasets considered here, both in the clean and in the noisy-retraining mode, while keeping the total runtime manageable. For each noise level with retraining enabled, a separate run is executed so that the classifier parameters match the target channel.

## 6. Results and Analysis

All experiments use the same 4-qubit variational circuit, the same depolarizing-noise grid p∈{0,0.02,0.05,0.10,0.20,0.30}, and the same four-path evaluation protocol: A (clean→clean), B (clean→noisy), C (noisy→noisy), and D (noisy→clean). For every nonzero *p*, the corresponding classifier is retrained with the matched noise channel enabled. Unless otherwise stated, results are reported as mean ± standard deviation over five independent random seeds. All attacks use perturbation budget ε=0.3 in the encoded angular feature space. We report results on four datasets of increasing difficulty: two-moons, breast cancer, Pima Indians Diabetes, and MNIST binary.

Because ASR is computed only over correctly classified test samples, we first report the clean and noisy accuracies and the corresponding number of correctly classified samples at each noise level. This table makes the ASR denominators explicit and helps distinguish changes in adversarial robustness from changes in standard classification accuracy under noise.

Table 4 shows that the number of correctly classified samples remains relatively stable across the tested noise levels for the three datasets. Therefore, the ASR trends reported below are not solely explained by a collapse of standard classification accuracy under strong noise.

### 6.1. Effect of Depolarizing Noise on Attackability

Table 5 shows the full Fast Gradient Sign Method results on the two-moons task; the clean quantum model is not trivially fooled (ASR-A =0.043 at p=0). A very small amount of depolarizing noise, however, makes the classifier most vulnerable: at p=0.02 all four paths lie between 0.106 and 0.147. Increasing *p* further does not monotonically increase robustness; the ASR stays in the 0.095–0.142 band.

The same experiment on the breast cancer dataset (Table 6) leads to a different picture. On the clean simulator the model is easier to fool, but as soon as p≥0.1 all four paths drop to about 5%, and at p=0.3 the ASR is practically zero. Here, inference noise works as an effective defense.

On the PID dataset the situation is more extreme (Table 7): at p=0.05 all four paths exceed 0.50 (A =0.508, B =0.516, C =0.516, D =0.528), so half of the test set can be flipped by a single-step attack under matched noise. MNIST (0/1) sits between those two: with p=0 we obtain ASR-A =0.408, but once *p* reaches 0.2 it drops to about 0.064. Altogether, this section shows that depolarizing noise does not have a uniform effect: for some tasks (breast cancer, MNIST) there is a clear robustness threshold around p≈0.15–0.2, while for others (two-moons, PID) a low-noise band actually maximizes ASR.

Furthermore, these tables reveal a finding: attacks generated under moderate noise often exhibit enhanced transferability (Path D) back to the clean model, sometimes exceeding the baseline attack (Path A). This is most evident on the PID dataset (Table 7). A similar effect is visible on the Moons dataset (Table 5), where at p=0.05, ASR-D (0.133) also surpasses ASR-A (0.115). This suggests that noise-aware generation does not simply overfit to the noise, but can find more generalized adversarial directions.

### 6.2. Impact of Attack Variants

As revealed in Section 6.1, the PID dataset exhibits the most significant and complex vulnerability, with its peak ASR exceeding 0.5, a fragility far greater than that observed for the BC and Moons datasets. We therefore concentrate our subsequent analysis on the different attack variants against the PID dataset to investigate the origin of this extreme vulnerability.

**Figure 3:**
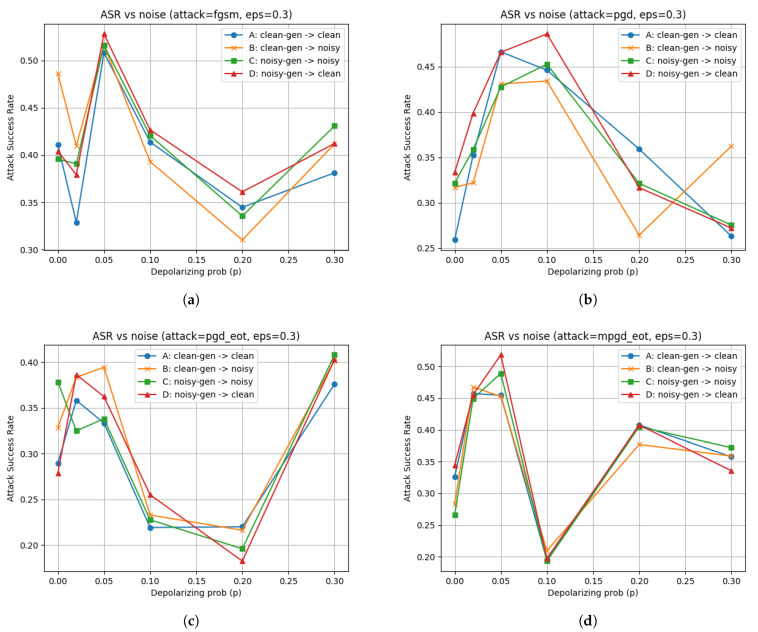
ASR versus depolarizing probability *p* on the PID dataset for four attack variants: (**a**) FGSM; (**b**) PGD; (**c**) PGD-EOT; and (**d**) momentum PGD-EOT. Each panel reports the four evaluation paths (A: clean→clean, B: clean→noisy, C: noisy→noisy, D: noisy→clean). All attacks exhibit a noise-dependent peak at small *p*, and in several cases Path D surpasses Path A.

To verify that the observed noise-dependent behavior is not specific to a single attack, we evaluate four gradient-based attacks on the same noise grid: FGSM, standard PGD, PGD with Expectation over Transformation (PGD-EOT), and momentum PGD-EOT. Figure 3 summarizes the results on the PID dataset, reporting ASR for all four evaluation paths.

From Figure 3 we make the following observations.

First, all four attacks are clearly noise-sensitive: none of the curves is flat in *p*, and the ASR can change by a factor of two over the tested noise range.

For one-step FGSM the best performance appears at a small depolarizing probability (p=0.05), while for standard PGD the maximum is reached slightly later (p=0.10); both support the existence of a low–moderate vulnerable noise band on this dataset.

Second, the EOT-based variants (PGD-EOT and momentum PGD-EOT) show a characteristic dip at p=0.10 but partially recover at higher noise levels (p=0.20–0.30), which means that EOT is effectively averaging over the noisy circuit and keeping the attack viable where plain PGD would degrade.

Third, across several noise levels we observe ${\mathrm{ASR}}_{D}\geq {\mathrm{ASR}}_{A}$, indicating that adversarial examples generated on a noise-aware model can transfer back to the clean model as well as, and sometimes better than, those generated on the clean model itself, but the effect should not be treated as a consistent property.

Together, these observations show that the phenomena reported in this section are not artifacts of a particular attack heuristic, but persist across single-step, iterative, and EOT-based attacks.

### 6.3. Cross-Noise Transferability

To evaluate how sensitive the attacks are to a mismatch between the noise assumed by the attacker and the actual inference noise, we produce 6×6 transferability matrices for the two-moons, breast cancer and PID experiments.

**Figure 4:**
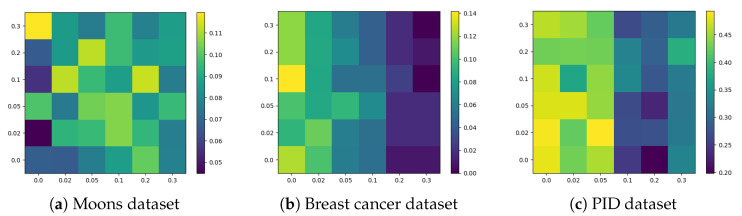
Cross-noise transferability matrices for PGD-EOT with ε=0.3. Rows correspond to the noise level used during attack generation, $p_{\mathrm{gen}}$, and columns correspond to the noise level used during inference, $p_{\mathrm{infer}}$. Cell colors indicate mean ASR over five random seeds, and the shared color bar gives the ASR scale. (**a**) Two-moons exhibits non-trivial off-diagonal transfer at a lower ASR scale. (**b**) Breast cancer is dominated by inference-side noise; high inference noise suppresses transfer. (**c**) PID shows a wide vulnerable band at low/medium inference noise.

As shown in Figure 4a, mismatched noise does not necessarily eliminate transferability on the two-moons dataset. Several off-diagonal entries remain nonzero, indicating that adversarial examples generated under one depolarizing probability can still transfer to models evaluated under a different noise level.

By contrast, Figure 4b shows that cross-noise transferability on the breast cancer task is largely governed by the inference-side noise level. The strongest transfer appears at low inference noise, especially for $p_{\mathrm{infer}}\in \left\{0.02,0.05\right\}$, where several generation noise levels yield ASR around 0.15–0.21. Once the inference noise increases to $p_{\mathrm{infer}}\geq 0.2$, the transferability is strongly suppressed, with most entries falling close to zero.

- Two-moons: The transferability matrix has a uniformly low ASR scale, with most entries around 0.04–0.07. Several off-diagonal entries are comparable to or slightly higher than the diagonal entries, indicating that exact matching between $p_{\mathrm{gen}}$ and $p_{\mathrm{infer}}$ is not essential on this task. However, the absolute ASR remains low, so the effect should be interpreted as weak but nonzero cross-noise transfer.
- Breast cancer: Transferability is mainly governed by the inference-side noise level. The brightest columns occur at low inference noise, especially $p_{\mathrm{infer}}\in \left\{0.02,0.05\right\}$, where ASR reaches roughly 0.14–0.21 for several generation noise levels. When $p_{\mathrm{infer}}\geq 0.2$, transfer is strongly suppressed and most entries are close to zero.
- PID: The matrix shows the highest overall transferability among the three datasets. A broad vulnerable region appears for $p_{\mathrm{infer}}\in \left\{0,0.02,0.05\right\}$, where many entries remain around 0.42–0.49. Although transfer decreases at larger inference noise, it does not vanish: even at $p_{\mathrm{infer}}=0.3$, several attacks retain ASR above 0.31. This indicates that the PID classifier remains vulnerable to noise-aware adversarial examples across a wide range of noise mismatches.

### 6.4. Dataset-Level Summary

Table 8 summarizes the main dataset-level patterns observed from the four-path FGSM results and the cross-noise transferability matrices.

### 6.5. Limitations and Practical Relevance

The experiments in this work are intended as a controlled proof-of-concept study rather than a direct evaluation of deployed quantum-security systems. The empirical setting is deliberately constrained: we use small simulated 4-qubit variational quantum classifiers, classical benchmark datasets compressed or mapped to four-dimensional angle-encoded inputs, and an independent depolarizing channel inserted immediately before measurement. These choices make it possible to systematically separate clean and noisy attack generation, clean and noisy inference, noise-matched retraining, and cross-noise transferability across multiple seeds.

Real systems may involve larger circuits, structured quantum data, task-specific measurement schemes, correlated noise, gate-dependent errors, calibration drift, readout noise, and hardware constraints that are not captured by the present simulator. The practical relevance of our results is therefore methodological: the study shows that noise should be treated as an explicit component of adversarial evaluation, and that attack-generation noise and inference noise should be separated when assessing robustness. Extending this protocol to larger circuits, realistic security-oriented datasets, richer noise models, and real quantum hardware is necessary before drawing deployment-level conclusions.

## 7. Conclusions

Within the constrained simulator setting studied here, depolarizing noise in a small variational quantum classifier does not provide a uniform robustness margin against adversarial examples. Using a noise-aware four-path protocol to separate generation noise from inference noise, we found that (i) the defensive effect of noise is strongly task-dependent. While some datasets become robust at high noise, others remain highly vulnerable even at p=0.3. We found that maximum vulnerability often occurs in a low-to-medium noise band (p≈0.05), directly contradicting the noise-as-a-defense intuition. (ii) Attackers that optimize through the noisy circuit via EOT-style attacks can recover much of the apparent robustness, confirming that noise-aware adaptation is a necessary component of the threat model. (iii) Noise-aware generation can sometimes produce perturbations that transfer back to the clean model as well as, or better than, clean-generated perturbations. This effect is most visible in some PID settings. These observations suggest that future evaluations of quantum models for security-sensitive applications should explicitly separate attacker-side and inference-side noise assumptions, rather than treating a single noisy configuration as sufficient evidence of robustness.

A limitation of the present study is that all results are obtained on small simulated VQCs with four-dimensional classical inputs and measurement-before-readout depolarizing noise. They should therefore be interpreted as evidence about this controlled evaluation protocol, not as a general characterization of all realistic NISQ hardware noise processes.

Future work could extend this to deeper circuits, other noise channels such as amplitude damping or correlated noise, real hardware platforms, and a systematic sensitivity analysis over multiple perturbation budgets. Subsequent work can complete these experiments, further consider certified robustness for quantum models in noisy settings, and embed the evaluation into automated QML robustness benchmarks.

## Figures and Tables

**Table 1:** Datasets and preprocessing used in the experiments. Explained variance is reported as mean ± standard deviation across five random seeds when PCA is used.

Dataset	Samples	Preprocessing	Explained Variance Retained
Moons	400	standardize; zero-pad → 4; map to [−π,π]	N/A
Breast cancer (BC)	569	standardize; PCA → 4; map to [−π,π]	0.831±0.003
Pima diabetes (PID)	768	standardize; PCA → 4; map to [−π,π]	0.802±0.004
MNIST (0 vs. 1)	∼1400	standardize; PCA → 4; map to [−π,π]	0.703±0.07

**Table 2:** Quantum model and noise configuration.

Item	Setting	Notes
Number of qubits	4	—
Encoding	single-qubit $R_{Y}\left(x_{i}\right)$	one feature per qubit
Variational block	StronglyEntanglingLayers	hardware-efficient pattern
Noise model	depolarizing channel with prob. *p*	applied before measurement
Measurement	$\langle Z_{0}\rangle$	mapped to label
Noise levels	p∈{0,0.02,0.05,0.1,0.2,0.3}	all reported in Section 6

**Table 3:** Adversarial attack settings.

Attack	ε	Steps	EOT *K*	Notes
FGSM	0.3	1	–	sign gradient, no EOT
PGD	0.3	10	–	step size α=0.02
PGD-EOT	0.3	10	16	α=0.02, noise-aware
Momentum PGD-EOT	0.3	10	16	α=0.02, momentum μ=0.9

**Table 4:** Clean and noisy accuracy at each depolarizing probability *p*. Values are reported as mean over five random seeds. Correct counts denote the mean number of correctly classified test samples used to define the ASR denominator.

Dataset	*p*	Clean Acc.	Noisy Acc.	Clean Correct	Noisy Correct
Moons	0.00	0.812	0.812	97.4	97.4
Moons	0.02	0.812	0.808	97.4	97.0
Moons	0.05	0.810	0.810	97.2	97.2
Moons	0.10	0.808	0.812	97.0	97.4
Moons	0.20	0.810	0.808	97.2	97.0
Moons	0.30	0.813	0.810	97.6	97.2
Breast cancer	0.00	0.784	0.781	127.0	126.4
Breast cancer	0.02	0.782	0.788	126.6	127.6
Breast cancer	0.05	0.781	0.773	126.4	125.0
Breast cancer	0.10	0.780	0.781	126.2	126.4
Breast cancer	0.20	0.770	0.762	124.6	123.2
Breast cancer	0.30	0.750	0.746	121.2	120.4
PID	0.00	0.631	0.641	145.8	148.0
PID	0.02	0.633	0.638	146.2	147.4
PID	0.05	0.635	0.636	146.6	147.0
PID	0.10	0.642	0.641	148.4	148.0
PID	0.20	0.647	0.651	149.4	150.4
PID	0.30	0.635	0.625	146.8	144.4

**Table 5:** FGSM (ε=0.3) on the two-moons dataset. Values are reported as mean ASR ± standard deviation over five random seeds.

*p*	ASR-A	ASR-B	ASR-C	ASR-D
0.00	0.043±0.019	0.060±0.018	0.045±0.019	0.043±0.016
0.02	0.147±0.018	0.124±0.019	0.113±0.011	0.106±0.017
0.05	0.115±0.016	0.106±0.017	0.113±0.025	0.133±0.021
0.10	0.086±0.027	0.124±0.018	0.142±0.021	0.104±0.028
0.20	0.125±0.016	0.114±0.019	0.105±0.018	0.124±0.010
0.30	0.115±0.018	0.095±0.016	0.132±0.022	0.123±0.021

**Table 6:** FGSM (ε=0.3) on the breast cancer dataset. Values are reported as mean ASR ± standard deviation over five random seeds.

*p*	ASR-A	ASR-B	ASR-C	ASR-D
0.00	0.160±0.044	0.193±0.045	0.186±0.035	0.196±0.044
0.02	0.114±0.035	0.122±0.028	0.145±0.046	0.139±0.068
0.05	0.112±0.067	0.121±0.068	0.095±0.054	0.144±0.043
0.10	0.053±0.029	0.096±0.054	0.079±0.042	0.053±0.028
0.20	0.045±0.056	0.036±0.042	0.045±0.047	0.036±0.060
0.30	0.018±0.043	0.019±0.029	0.027±0.030	0.018±0.041

**Table 7:** FGSM (ε=0.3) on the PID dataset. Values are reported as mean ASR ± standard deviation over five random seeds.

*p*	ASR-A	ASR-B	ASR-C	ASR-D
0.00	0.454±0.117	0.426±0.076	0.436±0.153	0.420±0.153
0.02	0.402±0.051	0.410±0.028	0.391±0.022	0.487±0.041
0.05	0.508±0.077	0.516±0.058	0.516±0.091	0.528±0.063
0.10	0.406±0.054	0.393±0.028	0.421±0.047	0.432±0.064
0.20	0.345±0.096	0.310±0.095	0.336±0.097	0.371±0.105
0.30	0.381±0.069	0.412±0.090	0.431±0.062	0.412±0.068

**Table 8:** Summary of the main observations across datasets.

Dataset	Four-Path ASR Pattern	Cross-Noise Pattern	Main Interpretation
Two-moons	Low overall ASR with non-monotonic changes across *p*.	Off-diagonal transfer remains nonzero but weak.	Noise reshapes vulnerability, but the effect is limited in magnitude.
Breast cancer	ASR decreases clearly at larger *p*, especially for p≥0.10.	Transfer is mainly suppressed by high inference-side noise.	This is the clearest case where depolarizing noise acts as an apparent passive defense.
PID	Highest ASR among the reported datasets; Path D exceeds Path A for all nonzero *p* under FGSM.	Transfer remains comparatively strong across several noise mismatches.	This dataset shows the strongest evidence that noisy-generation attacks can transfer back to the clean model.

## Data Availability

The data presented in this study are available in the article.
